# Divide and Conquer: Phenotypic and Temporal Heterogeneity Within CD8^+^ T Cell Responses

**DOI:** 10.3389/fimmu.2022.949423

**Published:** 2022-07-15

**Authors:** Arianne C. Richard

**Affiliations:** Immunology Programme, The Babraham Institute, Cambridge, United Kingdom

**Keywords:** CD8 T cell, heterogeneity, stochasticity, TCR - T cell receptor, T cell differentiation and function, cytotoxic T lymphocyte (CTL )

## Abstract

The advent of technologies that can characterize the phenotypes, functions and fates of individual cells has revealed extensive and often unexpected levels of diversity between cells that are nominally of the same subset. CD8^+^ T cells, also known as cytotoxic T lymphocytes (CTLs), are no exception. Investigations of individual CD8^+^ T cells both *in vitro* and *in vivo* have highlighted the heterogeneity of cellular responses at the levels of activation, differentiation and function. This review takes a broad perspective on the topic of heterogeneity, outlining different forms of variation that arise during a CD8^+^ T cell response. Specific attention is paid to the impact of T cell receptor (TCR) stimulation strength on heterogeneity. In particular, this review endeavors to highlight connections between variation at different cellular stages, presenting known mechanisms and key open questions about how variation between cells can arise and propagate.

## Introduction

The mammalian immune system relies on the generation of diverse immune cell types and subsets by developmental and differentiation programs. Cells undergoing such transitions pass through an intermediate zone, exhibiting further diversity on a continuum of changing characteristics. Once meeting the phenotypic criteria of a specific cell type or subset, cells can still exhibit extensive heterogeneity among other features. Enabled by advances in single-cell genomics, live imaging, and fate-mapping technologies, the past decade has seen a surge in research aimed at understanding cellular heterogeneity itself. Single-cell genomics can measure variability in genome-wide molecular characteristics in thousands to millions of cells (1–3). Long-term live imaging enables investigators to monitor temporal changes within individual cells or clonal lineages over increasingly long time-frames (4). Finally, fate-mapping methods can track the progeny of individual cells, or populations marked by past expression of a particular gene, to reveal diversification that occurs across many generations (5). These technologies have revealed that despite extensive variation between individual cells, means and variances of these heterogeneous populations can be remarkably stable (6, 7). This suggests that cellular heterogeneity itself is a regulated biological process.

**Figure 1:**
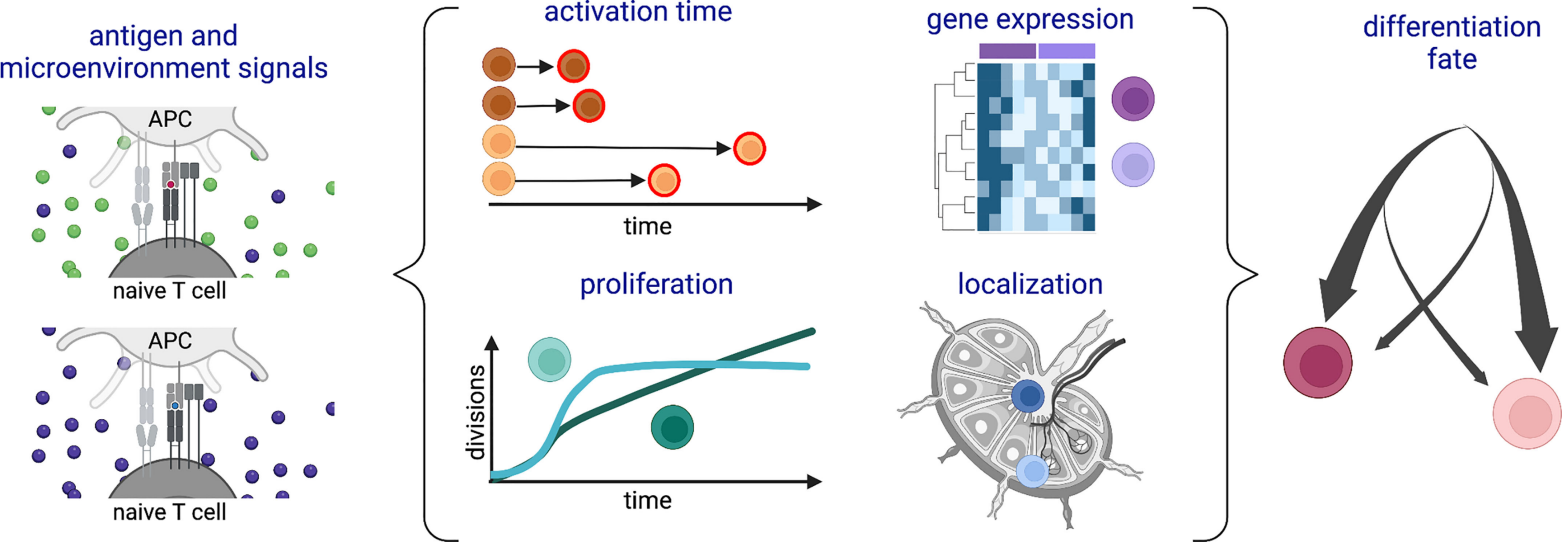
Schematic of types of heterogeneity among a population of CD8^+^ T cells responding to antigenic stimulation. Variation in (left) antigenic and/or microenvironmental signals is associated with (middle) different time delays before activation, gene expression patterns, proliferation profiles, and lymphoid tissue locations during the activation and expansion phases of the response. Many of these forms of heterogeneity are also associated with (right) subsequent differentiation fates. Created with Biorender.com.

Cells of the adaptive immune system, including CD8^+^ T cells, also known as cytotoxic T lymphocytes (CTLs), are unique in harboring hard-coded variation in the DNA sequences of their immunoreceptors. Although this can contribute to phenotypic diversity (8), it is not their only source of heterogeneity. This review will take a broad perspective to explore inter-cellular variation among CTLs at the levels of differentiation fate, response timing, gene expression, proliferation, localization and function (**Figure 1**), with a particular focus on how the strength of antigenic stimulation modulates this heterogeneity. Examples and connections between different forms of variation will be presented, highlighting outstanding questions about drivers and consequences of CTL diversity.

## The Branching Tree of Differentiation

Upon T cell receptor (TCR) stimulation with a peptide-MHC (pMHC) ligand on an antigen presenting cell (APC), a naïve T cell undergoes substantial metabolic and biosynthetic changes that initiate its proliferation and the differentiation of its progeny (9–11). The pool of activated, dividing cells that emerges during the first week of infection is already a heterogeneous mixture of both short-lived effector cells and memory precursors, which subsequently develop into various populations including stem-like, central, effector and tissue-resident memory (12). Chronic stimulation in the context of viral infection or cancer can then drive differentiation of additional subsets including exhausted or inflationary populations, which are themselves heterogeneous in nature (13, 14). High-dimensional single-cell measurements and fate-mapping techniques have been instrumental in revealing low-frequency but functionally important precursor cells that emerge during the early proliferative period [exemplified by (15–17)]. Beyond canonical differentiation pathways, single-cell transcriptomic studies of T cells from different anatomical locations, such as (18, 19), have demonstrated tissue-associated variation in gene expression within nominal subsets. Thus, T cell differentiation is a highly complex, diverging process.

Fate-mapping technologies have played a critical role in elucidating how the progeny of individual naïve CD8^+^ T cells populate diverse differentiated subsets (7). In 2007, Stemberger et al. transferred a single naïve TCR-transgenic T cell of known antigen specificity into a host and observed the differentiation of both effector and memory cells (20). Subsequently, another group used a lineage tracing system with genetic barcodes to track the progeny of individual naïve T cells and confirmed this model in which a single naïve cell can give rise to progeny with multiple differentiation fates (21). Such diversification of progeny has also been demonstrated in Th1 versus Tfh differentiation of CD4^+^ T cells (e.g (22, 23)). These data raised important questions about how such diversification is regulated. Are the proportions fixed? Are they consistent from one naïve cell to another? Three studies using different types of fate-mapping and limiting dilution cellular transfer systems clearly answered these questions: differentiation patterns emerging from each naïve T cell are highly diverse, but the population response to a specific challenge remains robust (24–26). This holds true even between naïve T cells with the same TCR, arguing against a deterministic mechanism whereby the TCR-ligand interaction programs a fixed pattern of differentiation fates. Instead, the fate distributions of individual cells were found to follow a probabilistic model (24).

## TCR-Ligand Interactions Can Skew Differentiation Fates

While differentiation fate distributions vary between individual naïve T cells, the population average from which these are sampled can be tuned by the nature of the stimulus. One such tuning factor is the strength of the TCR-ligand interaction. Two related metrics are frequently used to describe this interaction: “affinity”, which refers to the ratio between the on-rate and the off-rate, or in some cases simply the inverse of the off-rate, of each individual TCR-pMHC molecular interaction; and “avidity”, which describes the aggregate behavior of all interacting ligands and receptors and is thereby affected by additional factors such as ligand and receptor concentration. Several studies have investigated the impact of TCR-ligand interaction strength using modified influenza viruses, murine cytomegaloviruses (CMV) or *Listeria monocytogenes* (LM) strains expressing ligands of different affinities for the OT-I transgenic TCR (27–30). In these studies, naïve CD8^+^ T cells that received strong stimulation expanded more than weakly stimulated cells and preferentially differentiated into short-lived effector and effector memory populations. Accordingly, weakly stimulated naïve cells contributed fewer progeny to all differentiated subsets but were disproportionately found among memory populations, particularly central and tissue-resident memory. Similar results were observed in CD4^+^ T cells, with TCR-ligand interactions additionally impacting the balance of Tfh/Th1 or Th1/Th2 differentiation [exemplified by (31–34) and comprehensively reviewed in (35)]. Of note, one study that used modified LM infection to vary stimulation strength of OT-I CD8^+^ T cells observed no ligand-associated differences in the percentages of short-lived effector and memory precursor cells in the blood over a month of infection (36). This discrepancy may be due to differences in sampling sites and/or populations examined, but nevertheless it highlights the need for more work in additional model systems to understand the contexts in which stimulation strength impacts differentiation outcome.

The predicted consequence of differentiation biases driven by stimulation strength would be a pool of memory cells with greater TCR diversity and, on average, lower antigenic affinity than the pool of effector cells. Evidence of this phenomenon was observed in murine models of influenza infection (28, 37), while similar results were reported in CD4^+^ T cells during murine lymphocytic choriomeningitis virus (LCMV) infection (38). Interestingly, this latter study highlighted differences between TCR-ligand affinity and tetramer avidity measurements, serving as a reminder that additional factors such as TCR expression levels must be considered when comparing ligand binding tendencies between populations (38). Furthermore, exploration of a broader range of infections in murine and human systems will be important to understand the generalizability of antigen affinity differences between T cell subsets. Nevertheless, observations of greater TCR diversity within memory populations lead to the intriguing hypothesis that this serves as a mechanism to protect against reinfection with mutated pathogens. In support of this hypothesis, reducing clonal diversity of the memory pool by deleting *Cd27* (37) or interrupting EOMES/BLC2 signaling (28) impaired protection against mutated pathogen variants while leaving robust recall responses to the original pathogen. Thus, mechanisms that drive differences in TCR affinity and heterogeneity between effector and memory cells may have been naturally selected for their efficiency in fighting both current and future infections. It will be interesting to see whether future studies support or contradict this theory.

Chronic antigen presence can further drive CD8^+^ T cell differentiation. In certain contexts, such as latent CMV infection, CTLs with an effector memory phenotype and persistent effector functionality gradually expand in a process that has been termed memory inflation (14). Interestingly, while CTL expansion correlates with TCR-ligand affinity in acute infection (39, 40), this relationship appears to shift with latent infection. Using a model of murine CMV, a recent study found that while high affinity CTLs dominated early in the response, lower affinity cells became most abundant over time (41). These findings were corroborated by measuring the affinities of human CD8^+^ T cells specific for CMV-derived ligands, where inflation of the memory population was inversely correlated with its ligand affinity (41). The mechanisms underlying the inflation of low affinity populations during CMV infection are not completely resolved, but evidence suggests that they reflect early differentiation divergence that impacts the long-term self-renewing potential of each clone. Specifically, fate mapping experiments tracking the progeny of individual naïve murine CD8^+^ T cells showed that potential for long-term memory inflation was determined within 6 days of infection and correlated with a central memory precursor phenotype (42). Transcriptomic profiling of high-affinity CTLs shortly before the point at which they lost dominance in murine CMV infection revealed upregulation of co-inhibitory receptors, as well as a program of gene expression associated with senescence (41). Thus, the gradual evolution of ligand affinity may be the result of differentiation tendencies established in the early expansion phase of the T cell response.

In other contexts, such as chronic LCMV infection, continued antigen exposure leads to the development of an exhausted phenotype characterized by reduced effector function, sustained expression of coinhibitory receptors and altered cytokine and metabolic pathways (13). Coinhibitory receptors are rapidly expressed upon TCR stimulation, and studies altering the affinity or concentration of antigenic ligands found that strongly stimulated T cells expressed higher levels of co-inhibitory receptors within hours/days of activation, which dampened re-activation responses (43, 44). Elevated co-inhibitory receptor expression was also found to persist in CD8^+^ T cells over a month after vaccination with high affinity antigens (44). Recent work examining heterogeneity among exhausted CD8^+^ T cells in a murine model of chronic LCMV found that high affinity cells preferentially exhibited a terminal exhaustion phenotype, while lower affinity cells were more likely to fall into a cluster marked by expression of killer cell lectin-like receptors and cytotoxic genes (45). Thus, antigen affinity may also impact proliferative and self-renewal capacity settings of T cell exhaustion. In CD4^+^ T cells, divergent effects of TCR affinity were observed in chronic versus acute murine models of LCMV infection, such that strong stimulation biased cells toward Th1 differentiation during acute and Tfh differentiation during chronic infection (46). Interestingly, another study showed that the average affinity of CD4^+^ T cells responding to murine LCMV decreased over the transition from effector to memory-dominated responses, regardless of whether the infection was acute or chronic (38). Although not directly compared in CD8^+^ T cells, the results from acute infection, CMV, and LCMV described in this section suggest that CTL responses may also follow this pattern whereby shifts in clonal dominance depend, at least in part, on time rather than persistence of infection. Together, these data indicate that as T cells diversify through differentiation in response to an infection, the clonal heterogeneity of the responding population also undergoes reproducible, dynamic changes.

How heterogeneity within the context of naïve T cell activation might bias subsequent differentiation remains a key open question. The next three sections describe heterogeneity among early activation responses that might initiate such divergence.

## Diversification by Temporal Variation

Diversity in a pool of cells can arise through temporal variation of particular molecular changes. Accumulating evidence from many types of immune cells suggests that temporal variation can be governed by tunable probabilistic mechanisms (6). An exemplar of such a process is well-described in thymic development, where *Bcl11b* expression was found to be activated *via* an epigenetic switch with a long, stochastic time delay, which was itself tuned by histone methyltransferase/demethylase and transcription factor activity (47, 48). This work showcased the temporal heterogeneity that can emerge from rare, rate-limiting events (6). Altered biological conditions can then modify the population response by changing the probability distributions from which individual cells are sampled.

In mature T cells, a rate-limiting switch-like mechanism has been suggested for activation responses after TCR stimulation (49). Experiments using live imaging of individual TCR-ligand interactions found that T cells experienced a wide range of receptor-ligand dwell times, with only very long interactions or sequential, spatially co-localized interactions leading to T cell activation (50). As such events were rare, heterogeneity naturally arose. Moreover, the distribution of effective dwell times a cell might experience was found to be tuned by parameters such as the affinity of the TCR-ligand interaction (50). Accordingly, work in both naïve CD8^+^ T cells and effector CTLs has shown that TCR-ligand interaction strength modulates the mean and variance of time to response. Studies using single-cell RNA sequencing and mass and flow cytometry to examine naïve CD8^+^ T cell activation showed that strong stimuli drove more uniform, rapid initiation of activation events including signaling, transcription, cell division, and transcription factor nuclear localization, while weak stimuli induced responses with greater temporal variance and occurred, on average, later (51–55). In effector CTLs, live imaging demonstrated the same strength-dependent effects with respect to polarization of the centrosome and cytolytic granules toward the immunological synapse (56). All of these studies used transgenic T cells with ligands of known binding affinities. Extrapolating to physiological contexts with polyclonal T cell populations, their results suggest that the response times of individual cells are sampled from different distributions. Cells that activate at different times may then experience differences in repeat TCR engagement, cell-cell interactions or microenvironment composition during activation and/or expansion. As described below, all of these features have the capacity to propagate heterogeneity to subsequent stages of the T cell response.

## Heterogeneity of Gene Expression

The stimulation strength that a naïve T cell senses through its TCR, as well as costimulatory and cytokine receptors as discussed further below, may alter gene expression in the activating cell or its progeny. Studies in both CD4^+^ and CD8^+^ T cells found that, among cells that surpassed an activation threshold, stimulation strength correlated with expression of specific transcripts and proteins, often many hours/days after stimulation (52, 55, 57–64). Such effects may be modulated not only by transcriptional processes, but also post-transcriptional and translational mechanisms (9, 65). For example, weak TCR stimulation was found to be a poor driver of autocrine/paracrine IL2 production, which resulted in defective ribosome biogenesis and translation compared with strongly stimulated cells (64). How TCR-ligand affinity might initiate control over biosynthetic processes is not clear, but one possibility is that it relates to the duration of signals the T cell receives. The duration of T cell-APC interactions has been shown both *in vitro* and *in vivo* to correlate with stimulation strength [exemplified by (29, 44, 66–68)], and recent studies suggest that signal duration can impact gene expression. For example, use of a pharmacological intervention to interrupt TCR signals after different periods of time demonstrated different signal durations required for expression of early response transcription factors *Nr4a1* and *Nr4a3* (69). Likewise, experiments using an optogenetic construct to finely control patterns of receptor signaling in the Jurkat T cell line showed that transcriptional products persisted for a short time after signal interruption and accumulated with ongoing or repeated signaling (70). These data indicate that altering signal duration can lead to gene expression changes, which may propagate downstream. Accordingly, experiments manipulating the duration of TCR signaling showed differential effects on effector and memory populations subsequently differentiated *in vivo* (71–73). Thus, heterogeneity in antigen binding properties may impact differentiation *via* variation in experienced signal duration.

Differential gene expression can also be achieved by cellular division. Imaging of activating T cells at the point of mitosis demonstrated that sustained interactions with antigen-presenting cells could lead to asymmetric T cell division, wherein protein contents unequally segregated between daughter cells (74). In this and several subsequent studies, a bifurcation of gene expression was observed after the first cell division such that some daughter cells expressed genes associated with effector and others with memory cells, leading to the hypothesis that fate decisions are made at this first division stage (74–77). Accordingly, cells sorted by CD25 (IL2RA) and CD62L expression after the first division showed different memory recall phenotypes and capacity in adoptive transfer experiments (76). Altering stimulation strength was found to change the proportion of T cells asymmetrically dividing, thereby suggesting a means for affecting differentiation tendencies (29). However, it is unclear how the plethora of possible differentiation fates might be populated from a bifurcation at the first division, and other studies provide evidence for a contrasting model of differentiation fate segregation at a later time point. For example, fate-mapping experiments showed that cells expressing the short-lived effector cell marker KLRG1 during the early proliferative period were capable of differentiating into all types of memory cells (78). Likewise, experiments transferring CD8^+^ T cells to new hosts shortly after activation showed environmentally-controlled plasticity of fate distributions (79). Moreover, reports of fate-associated divergences in cellular division speed manifesting only after several rounds of replication (80–82) support a model of later segregation. In sum, additional lineage tracing work will be required to understand what role asymmetric division plays in directing CD8^+^ T cell differentiation programs.

## Proliferative Variability

Activating and differentiating T cells can exhibit highly heterogeneous proliferation behaviors. *In vitro* lineage tracing experiments found that the progeny of individual activated naïve CD8^+^ T cells divided a similar number of times but that variation existed between clones (83). Among a group of identically stimulated cells, the average expansion potential reflected TCR, costimulatory and cytokine signals (83, 84). These data suggest a mechanism to generate numerical heterogeneity among responding CTLs according to the signals each receives.

In a more complex *in vivo* environment, fate-mapping experiments found that the speed of division varied between expanding subsets, such that central memory precursors underwent a longer cell cycle than effector and effector memory precursor subsets (80). This work further demonstrated that the stimuli controlling cell cycle duration differed by subset, with effector cells responding to IL2 signaling and central memory precursors dependent on TCR stimulation. Subsequent *in vitro* long-term live imaging experiments, using anti-CD3 stimulation and culture with IL2 to promote sustained expansion, found rapid progression of all cells from division 2 through divisions 3 or 4, followed by a heritable split of division speeds (81). Faster divisions were associated with expression of the high affinity IL2 receptor component CD25, while slower divisions were associated with expression of CD62L (81), suggesting a relationship to the effector and memory precursor populations observed *in vivo* (80). A similar rapid initial proliferation, followed by a split in division times associated with differentiation marker genes, was also observed in a murine model of influenza infection using cell-cycle-phase reporter mice (82). The question thus arises whether differences in division time are causally related or consequential to divergent differentiation pathways. Experiments transferring slowly versus rapidly dividing T cells into new hosts two days after activation showed no difference in their intrinsic ability to generate memory cells (85). However, this time point is before the reported bifurcation of division times and thus may not have captured the fate-associated divergence if it exists.

Most recently, Bresser *et al.* developed a method for tracking division number in a population of cells by using a reporter construct with a synthetic short tandem repeat that has a fixed probability for slippage mutations at each division (86). In this system, the number of cell divisions corresponded to the fraction of cells in the population that expressed a fluorescent protein from the reporter construct. This work found that cells with a central memory phenotype generally underwent more divisions than those with an effector memory phenotype, but central memory cells also exhibited extensive heterogeneity of division history. Interestingly, central memory cells that had undergone fewer divisions proliferated more upon re-challenge. Thus, alongside division speed, it will be interesting for future work to test whether differences in generation number reinforce diverging differentiation pathways.

## Interplay of Environment With Response Heterogeneity

Variation in the experience of individual T cells can come not only from interaction with antigenic ligands but also each cell’s immunological context. This encompasses the physiological state of the host, the organ environment where the cell is located, and the microenvironment immediately surrounding the cell. Moreover, the relationship between a T cell’s environment and its response is bidirectional, with the nature of the response influencing the cell’s location and local milieu.

The inflammatory environment in which a naïve T cell is activated can impact its differentiation, skewing the distribution of precursor or differentiated subsets. Early work suggested that inflammation driven by live bacteria or chemical stimuli enhanced short-lived effector CTL responses while having relatively less influence on the generation of functional memory cells (87–89). This effect was found to be dependent, at least in part, on the cytokine IL12, which drove TBX21 (T-bet) expression in responding CTLs (88–90). Subsequent work demonstrated that IL2 could cooperate with inflammatory signals to promote short-lived effector differentiation (91, 92). This cooperation likely occurred not only at the level of signal integration but also as a feedback loop, with expression of CD25 dependent on inflammatory stimuli (80, 91). Beyond these two cytokines, comparisons of CD8^+^ T cell differentiation fates and their cytokine dependencies during different viral and bacterial infections demonstrated that the precise composition of the inflammatory environment altered the balance of differentiating effector and memory subsets (26, 93). One study tested the endurance and plasticity of environmental influences using TCR-transgenic CD8^+^ T cells capable of recognizing a shared antigen genetically added to vesicular stomatitis virus (VSV) and LM strains (79). Five days after infection, the authors transferred early effector cells that had yet to express KLRG1 or CD127 (characteristic of short-lived effector or memory precursor cells, respectively) into uninfected hosts and found differentiation patterns that recapitulated the cell subsets characteristic of the original infections, suggesting that the early inflammatory environment had lasting effects on differentiation programs. In contrast, when the authors transferred these early effector cells from VSV-infected into LM-infected hosts (and vice versa), differentiation patterns shifted to resemble those characteristic of the new host infection, indicating environmentally driven plasticity. Further work is needed to understand if this diversion was due to differential expansion or pathway plasticity at the individual cell level. Nevertheless, these data indicate that the inflammatory environment regulates the distributions of differentiating T cells during both the initial activation and expansion phases.

Within a given host environment, localization is also associated with cellular phenotype. For example, transcriptional divergence has been observed between CD8^+^ T cells of the same clonotype and nominal subset when extracted from different tissues (18). As T cells move between or within tissues, their cellular interactions and/or cytokine exposures change (94). Indeed, recent work found that cytokine availability was tightly regulated by proximity to producers and density of consumers, suggesting that subtle positional variation can change the signals received (95). Of course, the tendency to migrate to particular locations varies among a population of cells according to traits such as expression of chemokine receptors, as detailed below. Thus, microenvironmental interactions can be both a cause and consequence of divergent phenotypes.

**Figure 2:**
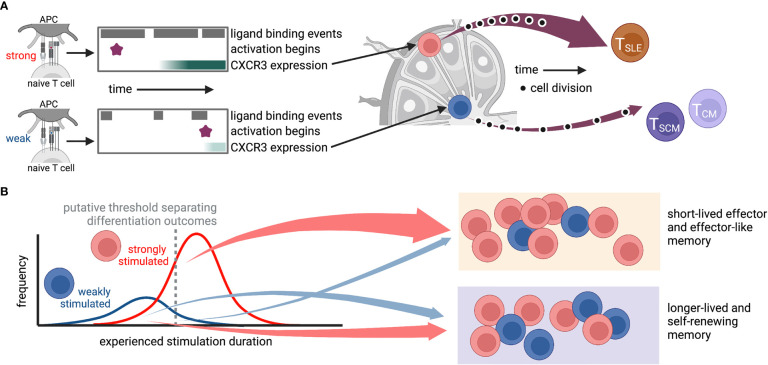
Possible route by which heterogeneity of TCR-ligand interactions could propagate through variations in stimulus duration, gene expression, and localization to diversify the T cell response. In this hypothetical situation, antigen affinity affects the frequency and duration of TCR-ligand interactions, the time at which the T cell activates, and expression of genes, including CXCR3. The level of CXCR3 expression then determines whether a cell traffics to the IFR or the middle of the lymph node, where it encounters niche-specific environmental cues that further promote specific differentiation programs. While cartoons in **(A)** depict a hypothetical “average” cell for each stimulus, those in **(B)** show putative cellular distributions for (left) experienced stimulation duration and (right) differentiation fate. This is only one of many possible routes that may connect ligand binding to differentiation outcomes. T_SLE_, short-lived effector; T_SCM_, stem-like memory; T_CM_, central memory. Created with Biorender.com.

Elegant studies manipulating chemotactic signals in CD8^+^ T cells have revealed the importance of CXCR3 signaling in directing activating cells to specific lymphoid tissue structures and promoting short-lived effector over memory differentiation (96, 97). Specifically, experiments using vaccinia virus infection found that CXCR3-deficient T cells were depleted from the marginal zone of the spleen, where the majority of inflammatory cytokines were expressed, and exhibited enhanced differentiation of memory precursors (97). Similarly, in LCMV infection, CXCR3-deficient T cells preferentially stayed in the lymph node paracortex instead of moving to the interfollicular regions (IFR) and showed differentiation divergence toward precursors of stem-like memory cells (96). Interestingly, TCR stimulation strength was found to be positively correlated with CXCR3 expression (67), as well as retention of T cells in the spleen (39) and IFR localization in the lymph node (67). It is therefore tempting to hypothesize a causal sequence whereby strong stimulation upregulates CXCR3, which directs cells to the lymph node IFR and provides an environment that drives effector differentiation (**Figure 2**). Future studies will be required to test this. Together, this work highlights how heterogeneity of T cell responses can propagate from variation in expression of a single chemokine receptor or subtle cell positioning all the way to differentiation fate.

Cellular interactions are governed by localization and form a critical part of the microenvironment that influences differentiation fate decisions. Early intravital imaging experiments immunizing with antigen-loaded dendritic cells (DCs) demonstrated that a single naïve TCR transgenic T cell makes multiple sequential APC contacts and that the duration of these interactions varies according to the activation stage of the T cell (98). As recently reviewed (94, 99), a large body of work over the past two decades has mapped the architecture of the lymph node and locations of innate and adaptive immune cells during an immune response. The majority of antigen presentation for naïve T cell activation is performed by conventional dendritic cells (cDCs). Type 1 cDCs reside primarily in the paracortex, express XCR1 and are specialized in antigen cross-presentation to engage CD8^+^ T cells (99). Intravital imaging studies demonstrated that cDC1s serve as a communication link between CD4^+^ and CD8^+^ T cells on the second day after infection, simultaneously engaging cells from both lineages and facilitating CD4^+^ T cell help to the CD8^+^ T cell response (100, 101). Inducible depletion of cDC1s disrupted clusters of antigen-specific CD4^+^ and CD8^+^ T cells, and skewed CD8^+^ T cell differentiation from memory toward effector pathways (100). Type 2 cDCs are often characterized by expression of CD11b (ITGAM) and tend to reside in the IFR and T cell-B cell border regions of the lymph node (99). While these DCs have been best described for their contribution to CD4^+^ T cell responses, it is speculated that they and nearby monocyte-derived dendritic cells contribute to the inflammatory signals that drive effector CD8^+^ T cell differentiation in the IFR (94, 96). In addition to DCs, studies of the mesenteric lymph node found that type 2 and type 3 innate lymphoid cells specifically reside in the IFR (102, 103), suggesting that they may also provide soluble and/or direct signals to activating T cells in this region. Moreover, recent studies have revealed an important role for stromal cell signals in directing the localization of immune cells (104), including CD8^+^ T cells (96). Additional evidence suggests that stromal cell interactions can directly impact T cell activation, metabolism and differentiation [e.g. (105, 106)], but these studies generally used *in vitro* activation and have revealed differences between mouse and human systems. Thus, more work is needed to understand the role of stromal interactions *in vivo*. Moving forward, the use of inducible model systems that can perturb gene expression within specific cellular populations at precise times during the lymph node response may shed additional light on how cellular interactions contribute to divergent differentiation pathways.

While we often think of CD8^+^ T cell responses being modulated by professional APCs, other innate immune components and CD4^+^ T cell help as described above, a series of studies has also demonstrated the importance of CD8^+^ T-cell-T-cell interactions and feedback in the course of activation. Direct interaction between activating T cells was observed 24 hours after in vivo stimulation in the form of ICAM1/LFA1-dependent T-cell-T-cell synapses (107). Subsequent work showed that these interactions costimulated paracrine IFNG (IFN-γ) signaling, which was associated with downregulation of CD25 and skewing away from effector toward memory cell differentiation (108). Feedback among activating T cells has also been found to occur *via* IL2 secretion. Multiple groups have identified instances of quorum sensing behavior and feedback loops for IL2 production within effector populations or mixed effector and regulatory T cell settings, particularly highlighting the emergence of robust population responses from highly heterogeneous expression and consumption among individual cells (109–112). Recent *in vitro* and mathematical modelling work built upon these findings to propose that T cells modulate IL2 according to cellular density *via* a series of nested feedback mechanisms involving CD28 and CTLA4 competing for CD80 and CD86 signaling (113). As cytokine signaling can impact differentiation outcomes, these data suggest that quorum sensing behavior might serve as a T-cell-intrinsic means of regulating differentiation. Accordingly, *in vitro* experiments in CD4^+^ T cells showed that higher cellular density led to an increased frequency of activated T cells expressing markers of memory precursors (114). The impact of T cell density on differentiation outcome in an *in vivo* setting is difficult to study as it has not yet been possible to alter local density without changing the frequency or baseline gene expression profiles of antigen-specific T cells. New experimental systems will be important for addressing this question.

Finally, as alluded to in multiple sections above, cytokine and costimulatory signals can feed into and amplify signaling networks initiated by the TCR, effectively enhancing the strength of stimulation a T cell experiences. Indeed, naïve cells are programmed to rely on these additional signals, as recently demonstrated by a study in which deletion of the RNA binding proteins ZFP36 and ZFP36L1 reduced dependence on CD28 signaling during early activation and enhanced effector differentiation (115). Investigations of signaling nodes responsible for conveying cytokine and costimulatory signals into the TCR activation network have identified elements of metabolic programming pathways, including PI3K/AKT and MYC (57, 60, 64, 116–119), consistent with extensive work showing that CD28 costimulation and IL2 signaling promote glycolytic metabolism (120). For example, *in vitro* co-culture experiments showed that IL2 produced by strongly stimulated T cells could push nearby, weakly stimulated cells over an activation threshold to initiate proliferation (116). This was blocked by treatment with LY294002 (116), which inhibits PI3 kinases, mTOR, and PIM kinases (119). Several other studies identified the transcription factor MYC, a key controller of metabolic reprogramming in activated T cells (121), as a biological node integrating TCR with costimulatory and IL2 signals (57, 60, 64, 117). Finally, THEMIS1, a signaling regulator that modulates SHP1 phosphatase activity, was found to be required for AKT/MYC pathway activation and proliferation induced by the addition of cytokines to weak TCR signals (118). In addition to direct effects on metabolic pathways, proteomics experiments revealed that IL2 also affected the metabolism of *in vitro*-derived effector CTLs by controlling expression of nutrient transporters and other environmental sensors (122). Given that metabolic shifts during T cell activation are strongly associated with differentiation outcomes (123), these data suggest a mechanism by which differences in the local cytokine and costimulatory environment during activation might propagate through divergent differentiation fates.

Taken together, the examples of environmental heterogeneity in this section reveal a complex interplay of antigenic and microenvironmental signals. These interactions may both reinforce (as in CXCR3-dependent migration) or restrain (as in IL2 quorum sensing) heterogeneity among responding CTLs to generate a diverse but robust response.

## Functional Diversity Beyond Differentiation

CTLs can perform a range of functions upon antigenic challenge, including secretion of cytolytic granules and cytokines such as IFNG, TNF (TNF-α) and IL2. Early work using a murine model of influenza infection demonstrated a hierarchy among cytokines secreted by CD8^+^ T cells, such that IL2-producing cells were a subset of those making TNF, which were themselves a subset of IFNG-producers (124, 125). In humans, examination of CD8^+^ T cells from HIV-infected patients revealed a set of frequently observed individual and combinatorial functions across single antigen-specific CD8^+^ T cells (126, 127). These studies found that the frequency of cells secreting multiple effector molecules (termed polyfunctional CTLs) correlated with reduced viral load. Association of CTL polyfunctionality with immune protection was subsequently observed in other contexts [e.g. vaccinia virus immunization (128), anti-CTLA4 cancer immunotherapy (129), and COVID-19, where polyfunctionality was highest in moderate compared with mild or severe cases (130)]. Much functional diversity is likely attributable to differentiation state (131, 132). However, a recent study that profiled the transcriptome and proteome of CTLs sorted according to IFNG and IL2 expression found molecular correlates of functional properties that were shared across multiple effector and memory subsets (133). These results suggest further functional tuning beyond differentiation outcomes that is regulated by specific molecular programs.

The regulation of functional diversity is not well-understood. Early studies of viral infection in mice and humans found an association between polyfunctionality and antigen avidity such that cells capable of multiple effector functions were more likely to strongly bind antigen (124, 127). Likewise, in *in vivo* rechallenge, memory cells derived from high affinity initial stimuli were more likely to express effector molecules than those initially stimulated with low affinity ligands (30). However, the differentiation trajectories of CTLs sampled in these studies were unclear. Experiments specifically altering ligand binding affinity during *in vivo* differentiation revealed little impact on the expression of key effector molecules among differentiated effector (39) and tissue resident memory cells (27), However, genome-wide transcriptional measurements in the latter study found considerable affinity-associated differential expression, leaving the door open to more subtle functional regulation (27). The context in which antigenic challenge occurs may also impact functional diversity, as exemplified by a recent study that found substantial gene expression differences in tissue-resident memory cells engaged by hematopoietic versus non-hematopoietic APCs during secondary infection (134). Thus, further work is needed to understand the drivers of functional heterogeneity within differentiated CTL populations.

## Origins of Heterogeneity Among Naïve T Cells

**Figure 3:**
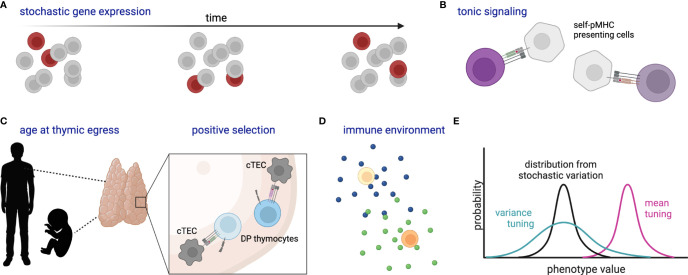
Schematic of stochastic and deterministic sources of naïve T cell heterogeneity. **(A)** Stochastic gene expression bursting within a population over time, where dark red cells indicate those cells randomly expressing the gene of interest at each time point. **(B)** Varied self-pMHC binding of naïve T cells affects gene expression and response characteristics. **(C)** Likewise, organism age at thymic egress (particularly adult versus fetus) and the strength of self-pMHC interaction during positive selection affect gene expression and response characteristics of peripheral naïve T cells; cTEC, cortical thymic epithelial cell; DP, double-positive. **(D)** Cytokines and other environmental components tune naïve cell reactivity. **(E)** Cartoon histograms depict the probability of a cell exhibiting a certain phenotype (e.g. expression of CD8 or a particular metabolic state). Stochastic variation as in **(A)** creates a distribution (black) which might then be tuned in variance (green) and/or mean (magenta) by additional factors such as **(B–D)**. All cells represent naïve T cells unless otherwise specified. Created with Biorender.com.

Observed differences in activation time and differentiation fate among naïve T cells with the same TCR [e.g. (24, 25, 52)] raise the question of what, if any, molecular mechanism allows one cell to initiate a particular molecular program when another does not (**Figure 3**). One explanation is that this is governed by underlying stochastic variability in gene expression or activity profiles, such that one cell is randomly more poised than another to respond at that instant. Within a homogenous population, such variability can be generated by transcriptional “bursting”, whereby a gene switches between states of active transcription and inactivity in a manner that is not synchronized within a population (135). In a homogenous population whose only source of variation is stochastic bursting, the time-averaged gene expression of individual cells would be uniform. Finding bursting genes responsible for heterogeneous responses can be experimentally challenging due to the transience of expression changes. Use of single-cell genomic and live imaging technologies has recently accelerated our ability to define and describe gene expression variability and may provide a means to determine how stochastic variation contributes to differential responses within a pool of naïve cells (1).

In addition to variation in the expression of individual genes, coordinated gene expression modules may be associated with the speed and quality of responses. One of the earliest studies of naïve CD8^+^ T cell response heterogeneity found that increased expression of the coreceptor CD8 allowed cells to respond to reduced concentrations of antigen, while increased expression of the phosphatase SHP1 reduced the maximal percentage of cells responding (136). Interestingly, these two molecules were also co-regulated, such that T cell activation responses were allowed to vary but only within biologically defined limits. Later investigations in naïve T cells expressing high versus low levels of surface CD8 corroborated these findings and demonstrated additional differences in gene expression including cell cycle and pro-apoptotic genes (137). Other experiments sorting naïve cells by glucose uptake capacity or markers of protein synthesis also revealed association with responsiveness to TCR stimulation (53). Together, these results highlight that not only single proteins but rather whole cellular programs are heterogeneously expressed among naïve T cells.

One of the most extensively studied factors associated with naïve T cell response propensity is tonic signaling from weak interactions with self-pMHC ligands. While a full discussion of this topic has recently been published elsewhere (138), a few key studies, particularly in CD8^+^ T cells, will be highlighted. Tonic signaling can diversify the antigen-inexperienced T cell population both by driving differentiation of virtual memory cells (139) and by affecting the true naïve population. Some of the first self-pMHC interactions that a T cell makes occur during thymic positive selection. The strength of this interaction was found to be associated with expression of the negative TCR signaling regulator CD5 on mature single-positive thymocytes and peripheral T cells (140). Comparisons of CD5^high^ versus CD5^low^ naïve CD8^+^ T cells found differences in common gamma chain cytokine sensitivity (141) and transcription factor expression (142), and also revealed subsets of CD5^high^ cells that expressed effector-associated molecules such as CXCR3, XCL1, and TBX21 (142). In antigenic challenge, CD5^high^ naïve CD8^+^ T cells, and in particular CXCR3^+^CD5^high^ populations, expanded more than CD5^low^ cells (142). Investigations into the mechanism behind this enhanced proliferation revealed greater responsiveness to inflammatory cues but not enhanced antigenic pMHC binding (142). Further associations of tonic signaling and CD5 expression have been found with other features involved in T cell responsiveness, including expression of the phosphatases CD45 and PTPN2 (143, 144) and metabolic state (145). Moreover, a study in naïve CD4^+^ T cells demonstrated CD5-associated differences in chromatin accessibility (146), suggesting that self-pMHC signaling can cause long-lived reprogramming in naïve T cells. Intriguingly, heterogeneity of CD5 expression and associated variation in responsiveness has even been observed among T cells with the same TCR [e.g. (147)], suggesting that inherent self-pMHC binding affinity is not the sole driver of this type of heterogeneity. While most of this work was done in murine systems, examination of CXCR3 expression on human naïve T cells revealed similar results such that CXCR3 was associated with effector-like transcriptional characteristics and greater response to non-specific activation (148). Together these data indicate that the naïve T cell pool is deterministically poised for diverse responses upon antigenic challenge.

Changes in thymic selection over the life course can also generate diversity among naïve T cells. A comparison of the TCR repertoire of T cells passing positive selection in neonatal versus adult mice found that strongly interacting cells were preferentially selected in the young thymus, generating a pool of peripheral T cells that was more self-reactive and more likely to express high levels of CD5 (149). This difference in thymic selection was concordant with previously observed differences in neonatal versus adult T cell responses (150, 151). Recently, an elegant study used an *in vivo* time-stamping method to mark naïve CD8^+^ T cells that developed at different points in life (152). This study demonstrated that fetal-derived naïve cells were molecularly distinct from adult-derived cells. Moreover, they were more likely to become virtual memory cells, respond rapidly to cytokine and infectious stimuli, and differentiate into terminally differentiated effector cells upon infection in the adult. Importantly, this effect of animal age was independent of post-thymic time or lymphopenic state at the time of thymic egress. Thus, thymically-driven variation in naïve T cells appears to persist at the cellular level as the animal ages.

Another potential contributor to variability among naïve T cells is subtle differences in cellular experience that accumulate over time. Such a mechanism is indirectly supported by observations of greater gene expression heterogeneity upon stimulation of naïve CD4^+^ T cells from aged compared with young mice (153). Differences in the T cell microenvironment, including some of those described above, may play a role in such processes. For example, recent work found that the combination of self-pMHC reactivity and exposure to type I interferon signaling drove a subset of CD5^hi^ naïve CD8^+^ T cells to express LY6C1 (also known as Ly6C) and preferentially expand and differentiate into short-lived effector cells upon antigen challenge (147). Thus, variation in the individual environmental experience of each naïve cell, perhaps accumulated over a lifetime, can also drive response heterogeneity.

Finally, there is intriguing evidence of genetic control of gene expression variation, including among CD8^+^ T cell populations (154, 155). These human genetic studies found polymorphisms associated with the distribution of gene expression across cells within each individual. Such findings indicate that regulation of inter-cellular heterogeneity generated by stochastic or deterministic mechanisms may, in part, be encoded within an organism’s DNA. Analyses of expression variability in the innate immune system suggested that evolutionary pressures have constrained expression variability of intracellular machinery such as transcription factors and kinases/phosphatases, while allowing highly variable expression of secreted signaling mediators and their receptors (156). It will be interesting to see whether similar features are found in T cell responses, or whether variability in the adaptive immune system is governed by different selective pressures.

## Discussion

This review has taken a broad perspective on heterogeneity in order to bring together different forms of variability reported among CTLs. As suggested by many of the studies described above, it is highly likely that these forms of heterogeneity are related and propagate from one to another. For example, one can envisage scenarios whereby heterogeneous gene expression within naïve T cells and variable interactions with TCR ligands on antigen-presenting cells result in diverse activation times, experienced signal duration and gene expression profiles, which in turn lead to different cell-intrinsic feedback loops, cell-cell interactions and/or cytokine exposures during proliferation and differentiation, thereby skewing the differentiation fates and functional properties of the progeny of each naïve cell (e.g. **Figure 2**). Many of the findings discussed here suggest that while gene expression and cellular interactions be governed by stochastic processes forming the backdrop for these events is heavily impacted by the natural history of the cell. Thus, although not deterministic, cellular experience likely controls the probabilities that underly divergent T cell responses.

Many connections between distant steps in the sequence from T cell selection through effector and memory responses remain to be investigated. The development of technologies to track the natural histories of individual cells and their progeny, such as inducible CRISPR scarring, should allow testing of such relationships in future work. By better understanding the drivers and propagators of T cell response heterogeneity, we may begin to anticipate and take advantage of this variation to achieve desired T cell responses through therapeutic manipulation.

## Author Contributions

The author confirms being the sole contributor of this work and has approved it for publication.

## Conflict of Interest

The author declares that the research was conducted in the absence of any commercial or financial relationships that could be construed as a potential conflict of interest.

## Publisher’s Note

All claims expressed in this article are solely those of the authors and do not necessarily represent those of their affiliated organizations, or those of the publisher, the editors and the reviewers. Any product that may be evaluated in this article, or claim that may be made by its manufacturer, is not guaranteed or endorsed by the publisher.
